# Use of the CytoSorb adsorption device in MDMA intoxication: a first-in-man application and in vitro study

**DOI:** 10.1186/s40635-020-00313-3

**Published:** 2020-06-15

**Authors:** Corinna N. Lang, Michaela J. Sommer, Merja A. Neukamm, Dawid L. Staudacher, Alexander Supady, Christoph Bode, Daniel Duerschmied, Achim Lother

**Affiliations:** 1 Department of Cardiology and Angiology I, Faculty of Medicine, Heart Center Freiburg University, University of Freiburg, Hugstetter Str. 55, 79106 Freiburg, Germany; 2 Department of Medicine III (Interdisciplinary Medical Intensive Care), Medical Center, Faculty of Medicine, University of Freiburg, Freiburg, Germany; 3 Institute of Forensic Medicine, Medical Center, Faculty of Medicine, University of Freiburg, Freiburg, Germany; 4 Institute of Experimental and Clinical Pharmacology and Toxicology, Faculty of Medicine, University of Freiburg, Freiburg, Germany

**Keywords:** MDMA, Intoxication, Multi-organ failure, Adsorption

## Abstract

**Background:**

3,4-Methylenedioxymethamphetamine (MDMA, “ecstasy”) abuse is frequent, and overdosing might cause severe and eventually lethal multi-organ failure. To date, there is no causal therapy of MDMA intoxication and removal of MDMA from the circulation might be a reasonable measure to prevent adverse courses after overdosing. We present here first-in-man experience and in vitro data supporting a potential role of an adsorber device in severe MDMA overdosing.

**Results:**

We applied a CytoSorb adsorber device in a 21-year-old male presenting with severe MDMA intoxication and multi-organ failure, including neurological impairment, hyperpyrexia, rhabdomyolysis, oliguric renal failure, liver failure, and coagulopathy with disseminated gastrointestinal and intramuscular bleeding. Use of the adsorber device was associated with a decline in MDMA concentrations in serum from 540 to 140 ng/ml within the first 24 h, a decrease of interleukin 6 and myoglobin levels, and subsequent clinical improvement. The patient was discharged from hospital after restoration of organ function and full neurological recovery. Effective elimination of MDMA by the adsorber device could be confirmed in vitro, when the device lowered MDMA concentrations to non-detectable levels.

**Conclusions:**

We report here first-in-man experience and in vitro data showing the capacity of a CytoSorb adsorber device for MDMA removal. Early integration of CytoSorb use may enhance the management of severe MDMA intoxication, though we cannot prove whether clinical improvement was directly related to elimination of MDMA or beneficial effects on rhabdomyolysis, hyperinflammation, or liver failure. Our findings encourage further investigation of the CytoSorb adsorber device in a prospective study and to evaluate its use for other intoxications.

## Background

3,4-Methylenedioxymethamphetamine (MDMA, “ecstasy”) is among the top ten drugs leading to emergency presentations in the USA and Europe [1, 2]. Approximately 1.7% of the young adult population have used MDMA within the last 12 months [2]. Although the rate of adverse events is lower than in other drugs of abuse, courses of severe MDMA intoxication range from full recovery to fatal outcomes [3–8]. The risk of death from a single use of MDMA has been estimated up to 1:20,000 [1]. MDMA stimulates the release of serotonin, dopamine, and norepinephrine, leading to the desirable feelings of increased energy and euphoria. However, overdosing might cause coma, seizures, liver failure, and hyperthermia with rhabdomyolysis, renal failure, and disseminated intravascular coagulopathy, likely due to serotonin syndrome [1, 9]. The current therapy of severe MDMA intoxication is non-causal and largely limited to sedation, cooling, and treatment of organ failure.

Removal of MDMA from the circulation might be an appropriate measure to prevent fatal adverse events after overdosing. The CytoSorb® adsorption device (CytoSorbents Europe, Berlin, Germany) contains porous polymer beads that adsorb cytokines but also other molecules within the 5–55 kDa range, e.g., myoglobin, bilirubin, and several therapeutic drugs, in a concentration-dependent manner [10, 11]. The adsorber cartridge Cytosorb consists of polystyrene divinyl benzene copolymer beads with a biocompatible polyvinyl pyrrolidone polymer coating. The beads have a size of approximately 300–800 μm with pores of approximately 20–50 A. The adsorption device is increasingly used for different indications, including critically ill patients with sepsis or post-resuscitation syndrome [12–14]. We describe here first-in-man experience and provide in vitro data supporting a potential use of a CytoSorb adsorber device in MDMA intoxication.

## Results

### In-man application

A 21-year-old, previously healthy male (height 178 cm, body weight 56 kg) was admitted to the emergency department after MDMA, clonazepam, and pregabalin abuse. He presented with typical symptoms of severe MDMA intoxication including neurological impairment (Glasgow coma scale 6), mydriasis, hyperpyrexia (body temperature 40.3 °C), tachycardia (heart rate 167/min, blood pressure 98/61 mmHg), and dehydration. Shortly after arrival, he had tonic-clonic convulsive seizures requiring endotracheal intubation. Hypoglycemia was corrected and body temperature lowered to 39 °C by cold infusions and a single dose of paracetamol. A computed tomography scan did not reveal any cerebral pathology.

**Fig. 1:**
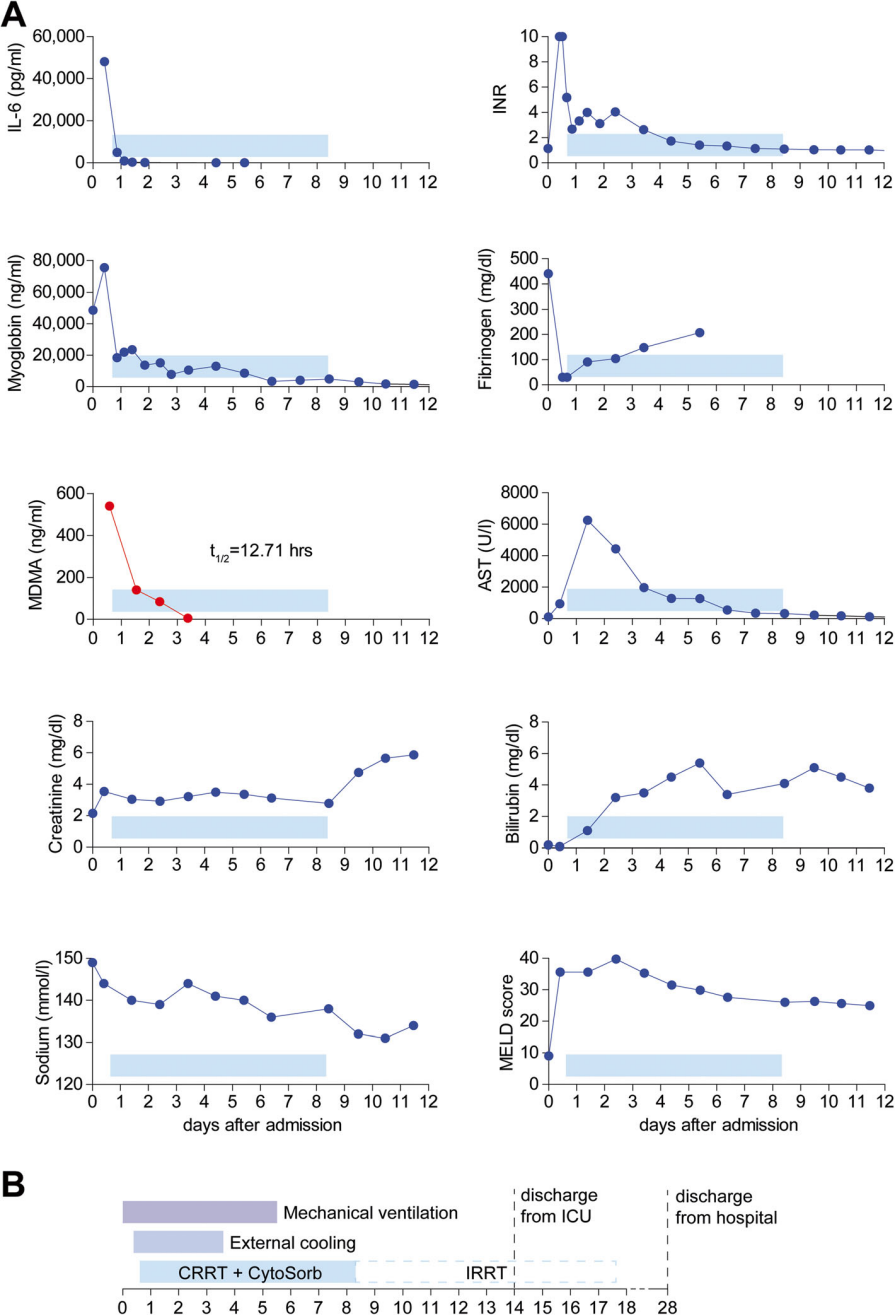
Time course of laboratory findings and treatment milestones during clinical application of the adsorber device after MDMA intoxication. Serum concentrations of interleukin 6 (IL-6), myoglobin, 3,4-methylenedioxymethamphetamine (MDMA), creatinine, sodium, bilirubin, and international normalized ratio (INR), fibrinogen levels, and aspartate amino transferase (AST) activity were repeatedly measured during the clinical course. MELD score (Model of End-Stage Liver Disease) was calculated to assess liver injury (**a**). Timeline indicating durations of mechanical ventilation (purple), external cooling (mid-blue), continuous renal replacement therapy (CRRT) with CytoSorb (light-blue), and intermittent renal replacement therapy (IRRT, dashed light-blue) (**b**)

After being transferred to the intensive care unit, he showed recurrent hyperthermia and rapidly developed massive rhabdomyolysis (myoglobin 75,420 ng/ml), oliguric renal (serum creatinine 3.54 mg/dl) and liver failure (MELD score 36, Fig. 1a) [15], and severe coagulopathy with disseminated gastrointestinal and intramuscular bleeding. During the first 15 h, plasmatic coagulation and fibrinogen levels decreased beyond the detectable range. Hemoglobin and platelet counts dropped by 70% (Hb 16.1 to 4.9 g/dl) and 73% (platelets 196 to 52 cells/nl), respectively. Transfusion of 3 red blood cell concentrates, 3 platelet concentrates, and 6 units of fresh frozen plasma; substitution of coagulation factors; and moderate vasopressor support were provided. Serum lactate increased to 4.3 mmol/l while fluid resuscitation using balanced crystalloid solution and albumin infusions was continued. Body temperature was maintained between 35.9 and 36.5 °C by a surface cooling device (ArticSun®, Medivance, Louisville, CO, USA). Twenty-four hours after hospitalization, calculated SAPS II score of 85 predicted an in-hospital mortality 95% [16].

First quantitative measurement of MDMA in blood serum 15 h after admission revealed a concentration of 540 ng/ml, confirming the suspected severe overdosing and suggesting an even higher peak concentration. Clonazepam (180 ng/ml) and pregabalin (660 ng/ml) probably contributed little to the symptoms. For anuric renal failure, we initiated continuous renal replacement therapy (Fresenius Medical Care, multifiltrate Ci-Ca CVVHD, Ultraflux AV 1000S, blood flow 80 ml/min, dialysate flow 1600 ml/h, mean effluent flow rate 30.1 ml/kg/h) starting 16 h after admission and included a CytoSorb adsorption device into the hemodialysis circuit to treat rhabdomyolysis and systemic inflammation. During the first adsorption interval of 24 h, myoglobin and interleukin-6 levels in serum quickly decreased by 76% (myoglobin 75,420 to 18,400 ng/ml) and 90% (IL-6 48,129 to 4991 pg/μl), respectively (Fig. 1a). Adsorption therapy was continued for 7 days at constant blood flow of 80 ml/min with replacement of the device every 24 h (7 devices in total).

Though CytoSorb therapy was primarily initiated to treat rhabdomyolysis and systemic inflammation, we hypothesized that potential elimination of MDMA by the CytoSorb may have added further benefit to the patient. Serial measurements revealed a decline in MDMA concentrations in serum from 540 to 140 ng/ml within the first 24 h after adsorption therapy was started. During 3 days, serum MDMA levels of the patient showed a one-phase exponential decay with a calculated half-life of 12.71 h (*R*^2^ = 0.990) despite persisting anuric renal failure (Fig. 1a). Within 36 to 48 h after admission, the patient’s condition improved significantly. Serum lactate normalized and vasopressor support could be discontinued. Increasing fibrinogen levels and decreasing aspartate transaminase activity in serum suggested overcome coagulopathy and recovery of liver function. External cooling and mechanical ventilation could be discontinued at day 4 and 6 after admission, respectively, and the patient left the intensive care unit at day 14. Renal dysfunction fully resolved after 17 days of renal replacement therapy (Fig. 1b). Increasing fibrinogen levels and decreasing aspartate transaminase activity in serum suggested recovery of liver function (Fig. 1a). MELD score continuously improved to 9 at day 27. The patient showed full neurological recovery and was discharged to rehabilitation therapy at day 28 (Fig. 1b).

### Elimination of MDMA using the CytoSorb in vitro

**Fig. 2:**
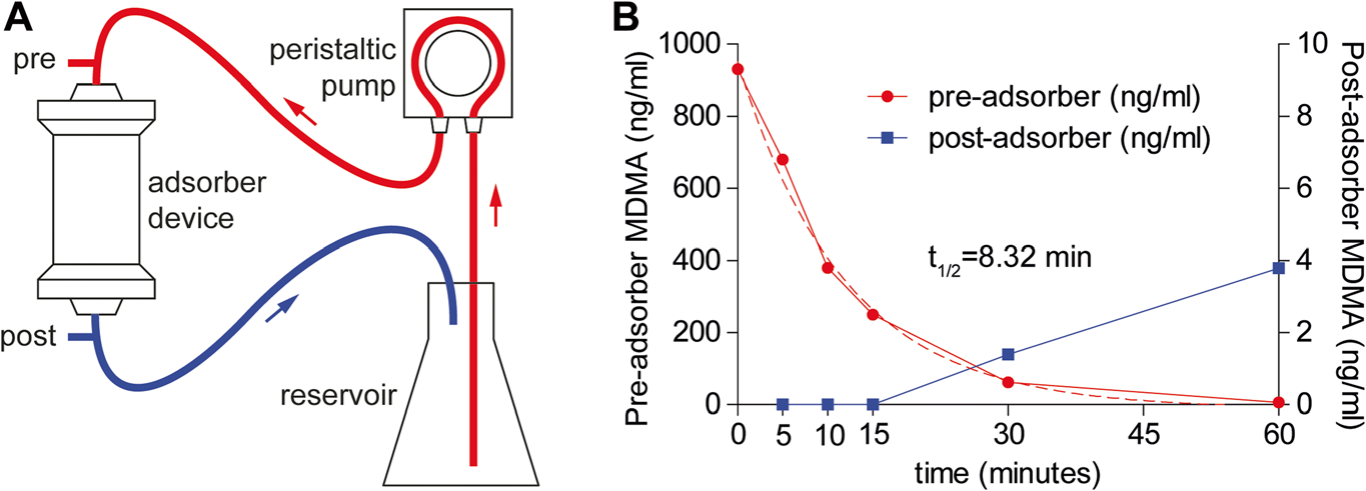
Adsorption of MDMA in vitro. Serum containing 3,4-methylenedioxymethamphetamine (MDMA-FCS, 1 μg/ml) was circulated from a reservoir at a flow rate of 170 ml/min (**a**). MDMA-FCS samples were taken from the circuit before (pre, red) and after (post, blue) the adsorber device at 0, 5, 10, 15, 30, and 60 min, and MDMA was quantified by HPLC (**b**). Dashed line indicates calculated half-life

Following our clinical findings, we evaluated the capacity of the CytoSorb adsorber to eliminate MDMA in vitro. MDMA was dissolved in FCS at a similar concentration that was observed in our patient and circulated in a custom-made system including a CytoSorb adsorber device. After 5 min, MDMA concentration measured distal of the adsorber device was non-detectable, indicating full removal of MDMA by the adsorber. Serial measurements showed a one-phase exponential decay of MDMA in the reservoir with a calculated half-life of 8.32 min (*R*^*2*^ = 0.993) (Fig. 2). Interestingly, after 30 and 60 min, we detected a slight increase in post-adsorber MDMA concentration (1.4 and 3.8 ng/ml) (Fig. 2).

## Discussion

We report here first-in-man experience and in vitro data showing the capacity of a CytoSorb adsorber device for MDMA removal both in a clinical scenario and in vitro. Our data suggest that CytoSorb use may be beneficial in patients with severe MDMA intoxication.

MDMA content of “street” ecstasy is highly variable and has been increased during the past decade, thus aggravating the risk for intoxication [1, 2]. The CytoSorb device contains adsorber beads that are specifically designed to remove molecules between 4 and 20 kD [17] but bind to a wide range of molecules. In this case, CytoSorb use was associated with a quick decrease in interleukin 6 and myoglobin levels, supporting the previously described beneficial effects of CytoSorb use on rhabdomyolysis, hyperinflammation, and liver failure [14]. Thus, CytoSorb use may be beneficial to attenuate organ damage secondary to MDMA intoxication. In addition, it has been shown that the CytoSorb device has a high binding capacity of substances of low molecular weight comparable to MDMA (193.2 D) including several therapeutic drugs, e.g., valproic acid, theophylline, and phenobarbital [11]. Due to a low volume of distribution of 4 to 7 l/kg [18] and protein binding of only 34–40% [19], there is a relevant proportion of free MDMA in blood. Additionally, the partially protonated basic nitrogen of MDMA (pKa 8.7) [20] may interact with the carboxylic acid amide of the pyrrolidone-coated surface of the beads. Therefore, an adsorption of MDMA on CytoSorb and subsequent removal seemed to be likely, but remained hard to predict. We show here that MDMA was effectively eliminated by the CytoSorb in vitro and its use may thus be promising after overdosing.

Other strategies for clearing inflammatory mediators in sepsis are undergoing clinical evaluation, including high-volume hemofiltration, cascade hemofiltration, plasmapheresis, or high cutoff hemofiltration, and may represent an alternative to CytoSorb [21, 22]. For example, it has been demonstrated that CRRT with a high cutoff hemofilter likewise facilitates interleukin 6 and myoglobin plasma clearance [23]. Whether this also applies to MDMA removal remains to be investigated.

Importantly, time course and severity of the undesired pharmacological effects of MDMA largely follow blood concentration course although the correlation of the effects with the absolute MDMA concentration is rather low, especially in multiple dosing [24]. After intake, gastro-intestinal absorption of MDMA is rapid and peak plasma concentrations are attained after approximately 2 h, which makes gastric lavage or charcoal use ineffective in most cases [1, 9]. This implies that adsorber therapy should be initiated as early as possible in suspected severe overdosing to remove as much of the compound as possible and potentially prevent organ damage.

Plasma half-life of MDMA in the patient described here was 12.7 h. In previous studies, mean plasma half-life of MDMA in healthy adults after controlled administration of a defined dose was 7–9 h [9, 18]. However, pharmacokinetics of MDMA is non-linear, and elimination is prolonged at higher concentrations due to saturation or inhibition of metabolizing enzymes. This effect was already evident at concentrations ranging from 163 to 292 ng/ml, but MDMA peak concentration in our case was at least 540 ng/ml [9, 18]. Data on the metabolism and renal clearance of MDMA in patients with liver and kidney failure, which was present in our patient, is not available, but the organ failure probably further impairs drug elimination and prolongs plasma half-life. Interestingly, in vitro, the post-adsorber MDMA concentration slightly increased after 30 min. The capacity of the adsorber depends on the amount of available sorbent, suggesting a beginning saturation of the adsorber device. Our finding is in line with previous studies when desorption of therapeutic drugs had been observed between 30 and 60 min of perfusion [11]. Importantly, this argues for an only moderate affinity of the adsorber to different drugs [11]. From a translational point of view, this finding suggests that frequent replacement of the adsorber device during clinical application could enhance efficacy and prevent desorption, but optimal replacement intervals remain to be investigated.

## Conclusions

In conclusion, integration of CytoSorb use may enhance the management of severe MDMA intoxication. Though our data suggest that the use of an adsorber device facilitates the elimination of MDMA, we cannot prove whether this was directly related to clinical improvement. However, when initiated early, MDMA removal may add on the beneficial effects of CytoSorb use on rhabdomyolysis, hyperinflammation, and liver failure. Further investigations are necessary to validate our observations in a prospective study and to prove a causal relationship between the use of an adsorber device and clinical outcome. In addition, our findings encourage systematic testing of CytoSorb use for other intoxications.

## Methods

### Patient selection and data acquisition

We retrospectively evaluated the application of a CytoSorb adsorber device in a case of severe MDMA intoxication. Data were extracted from medical records. Written informed consent was obtained from the patient for publication of this report. Statistical analysis was performed using GraphPad Prism 5.04.

### In vitro study

To directly evaluate the capacity of the CytoSorb hemoadsorption device (CytoSorbents Europe, Berlin, Germany) to eliminate MDMA, we modified a previously published protocol [11]. A CytoSorb device was integrated in a custom-made system applying a peristaltic pump (AlphaControl PSP-V12G, Fig. 2). MDMA (Sigma-Aldrich, Taufkirchen, Germany) was dissolved at a concentration of 1 μg/ml in 2.500 ml fetal calf serum (FCS, Sigma-Aldrich, Taufkirchen, Germany). The solution was circulated from a reservoir at a flow rate of 170 ml/min for 60 min. Samples (1 ml) were taken from the circuit simultaneously before (pre) and after (post) the adsorber device at 0, 5, 10, 15, 30, and 60 min. MDMA concentrations were analyzed by a validated liquid chromatography-tandem mass spectrometry method after solid-phase extraction (lower limit of detection 0.6 ng/ml, lower limit of quantification 0.8 ng/ml). MDMA half-life was calculated by a one-phase exponential decay model (*Y* = (Y0 − NS) × exp(− K × X) + NS) using GraphPad Prism 5.04.

## Data Availability

Not applicable

## Abbreviations

FCS: Fetal calf serum
MDMA: 3,4-Methylenedioxymethamphetamine
